# Research on the collaborative evolution process of information in public health emergencies based on complex adaptive system theory and social network analysis: a case study of the COVID-19 pandemic

**DOI:** 10.3389/fpubh.2023.1210255

**Published:** 2023-09-25

**Authors:** Kun Lv, Xingyu Luo, Jiaoqiao Shan, Yuntong Guo, Minhao Xiang

**Affiliations:** ^1^School of Business, Ningbo University, Ningbo, China; ^2^School of International Trade and Economics, University of International Business and Economics, Beijing, China

**Keywords:** epidemic response, collaborative networks, adaptive dynamics, social interactions analysis, pandemic management

## Abstract

**Introduction:**

This review aimed to elucidate the significance of information collaboration in the prevention and control of public health emergencies, and its evolutionary pathway guided by the theory of complex adaptive systems.

**Methods:**

The study employed time-slicing techniques and social network analysis to translate the dynamic evolution of information collaboration into a stage-based static representation. Data were collected from January to April 2020, focusing on the COVID-19 pandemic. Python was used to amass data from diverse sources including government portals, public commentary, social organizations, market updates, and healthcare institutions. Post data collection, the structures, collaboration objectives, and participating entities within each time slice were explored using social network analysis.

**Results:**

The findings suggest that the law of evolution for information collaboration in public health emergencies primarily starts with small-scale collaboration, grows to full-scale in the middle phase, and then reverts to small-scale in the final phase. The network’s complexity increases initially and then gradually decreases, mirroring changes in collaboration tasks, objectives, and strategies.

**Discussion:**

The dynamic pattern of information collaboration highlighted in this study offers valuable insights for enhancing emergency management capabilities. Recognizing the evolving nature of information collaboration can significantly improve information processing efficiency during public health crises.

## 1. Introduction

Public health emergencies pose a substantial threat to global health security, economic stability, and societal well-being. These events, marked by their sudden onset and unpredictability, require a proficient and comprehensive information collaboration mechanism to facilitate effective response strategies. The COVID-19 pandemic serves as a salient illustration of the crucial role of information collaboration and communication in managing public health crises. Italy, as an example, as one of the first countries outside China to experience a significant outbreak, found itself dealing with a swiftly escalating situation. The Italian healthcare system, reputed as one of the most robust in Europe, was quickly overwhelmed by the surge of patients, leading to severe resource and personnel constraints. The national response was significantly influenced by the quality and timeliness of information shared among a range of stakeholders. Government agencies, public health organizations, healthcare providers, and the general public collaborated, sharing data, resources, and responsibilities to manage the crisis. This scenario highlighted the essential role of information collaboration in public health emergencies management. Notably, this predicament was not confined to Italy; similar challenges were faced worldwide. Italy serves as a notable example, yet the dynamics and outcomes of information collaboration differ significantly among major nations, underscoring the critical importance of such collaboration in addressing public health crises. In the United States, a decentralized healthcare system combined with the politicization of the pandemic resulted in disparate approaches and levels of information-sharing across states. South Korea swiftly established a comprehensive information-sharing system among the government, medical institutions, and the public, ensuring quick contact tracing and testing. In contrast, the United Kingdom initially struggled with timely information dissemination before adopting a more centralized strategy for managing and disseminating pandemic-related data. In countries such as India, the immense and varied population posed challenges to consistent and transparent information dissemination, leading to diverse regional responses. A comparison of these distinct experiences from pivotal nations offers insights into the factors and consequences of information collaboration during global health crises. Thus, the diverse responses to COVID-19 globally provide instructive insights into the role of information collaboration in public health emergencies. From a theoretical perspective, information collaboration in such emergencies is viewed as a complex adaptive system, characterized by complexity, nonlinearity, and adaptability. It is thus hypothesized that this system will undergo an evolutionary process throughout the public health emergency. This theoretical framework forms the foundation for the ensuing analysis and guides the subsequent empirical investigation. The main objectives of this research are: (a) to analyze the evolutionary pattern of information collaboration in public health emergencies, (b) to reveal the internal dynamics and characteristics of the collaboration network, and (c) to deliver insights and recommendations for enhancing information collaboration efficiency during public health emergencies. Building on the case study of the COVID-19 pandemic, this research aims to elucidate these pivotal issues and contribute to the continuing discussion on information collaboration in public health emergencies. It is anticipated that this timely and relevant research will serve as a valuable resource for policymakers, healthcare professionals, and researchers in this domain.

## 2. Literature review

From the appearance of the Antonine Plague in AD 165 to the current COVID-19 pandemic, the war between humanity and epidemics has been ongoing throughout history. Due to the continuous occurrence of various sudden public health events, scholars have paid extensive attention to the information coordination of these emergencies (1). Information coordination plays a pivotal role in public health emergencies, affecting the efficiency and effectiveness of response measures (2). Recent studies have highlighted different aspects of this topic. For instance, Anita and Sukomal explored the role of social media platforms in disseminating information during crises, emphasizing the need for verified information from authoritative sources (3). On the other hand, the study by Balaji et al. underscores the importance of diversity in information coordination mechanisms, arguing for an adaptable and inclusive approach that caters to different regions and demographic groups (4).

In terms of method selection, Saura, Reyes-Menendez, and Palos-Sanchez illustrate how Twitter sentiment analysis can be effectively used to gauge public reaction to events such as Black Friday sales, a technique that could also be applied to public health crises (5). Similarly, Palos-Sánchez, Folgado-Fernández, and Rojas-Sánchez applied text and opinion mining techniques in analyzing the discourse surrounding virtual reality technology, demonstrating their usefulness in extracting actionable insights from large volumes of text data (6). Additionally, Saura, Palos-Sanchez, and Grilo used sentiment analysis through text data mining to identify indicators of startup business success, reinforcing the potential of these analytical methods in various research fields (7). As for methods, significant advancements have been made in the development and application of computational and analytical tools to enhance information coordination. Sarker demonstrated the use of machine learning algorithms in mining social media data for real-time tracking of public sentiment and misinformation spread during health crises (8). Furthermore, an emerging body of literature focuses on the application of complex adaptive systems theory in understanding and improving information coordination processes (9).

This study draws on these previous works to examine the evolution of information coordination in public health emergencies. The first aspect of this research is the feature analysis and definition of information coordination in sudden public health events. Waugh et al. (10) suggest that the information coordination of sudden public health events is a multi-party information dissemination process based on information flow, which includes four dimensions: information, information personnel, information environment, and information technology. Luo et al. (11) pointed out that emergency information for sudden events has three essential characteristics: speed (immediacy and real-time), quality (accuracy and reliability), and efficacy (applicability and value). Currier et al. (12) summarized the experience of responding to Hurricane Katrina and found that collaborative cooperation among various departments and multiple parties is necessary in the emergency management process. Park (13) argues for the necessity of information coordination in sudden public health events and believes that horizontal cross-organizational information coordination is advantageous for improving organizational output efficiency and flexibility in complex situations.

The second aspect of this research is the analysis and identification of problems in the information collaboration mode during public health emergencies. At the level of information subjects, Zhou (14) suggested that grassroots communities have limited capacity in information collaboration during public health emergencies, medical institutions are geographically dispersed with severe information technology deficiencies, resulting in the widespread existence of information islands. At the information environment level, Ramon et al. (15) summarized the influencing factors of information collaboration, including policies and institutions, management and organization, information and technology, and environment. For this purpose, Salmon et al. (16) studied the multi-agency coordination process during emergency response and emphasized that the coordination level between military and civilian organizations is crucial for the efficiency of multi-agency systems in responding to large-scale emergencies. They identified factors that affect information sharing such as untimely sharing of information, inaccurate and unreliable information, incomplete information, and unclear organizational responsibilities. Fan et al. (17) studied the cross-organizational information sharing and utilization mechanisms in joint emergency actions and suggested that a structurally sound cross-organizational network and a leadership department with good information accessibility are key factors that significantly affect the efficiency of emergency collaboration and the ability to absorb information. Lencucha et al. (18) proposed that information sharing is a key factor in effectively responding to infectious disease outbreaks, and the international coordination system largely depends on timely and accurate information provided by governments during health risks in epidemics. This information supports the decision-making process for declaring a public health emergency of international concern by the World Health Organization, as well as assisting the WHO in cooperating with governments to coordinate the containment of cross-border epidemics.

The third aspect of this research is the optimization of the mechanism of information collaboration during public health emergencies. Collaborative governance theory combines collaborative theory and governance theory, and Donahue et al. (19) suggested that “the essence of collaborative governance is that government organizations and non-government organizations actively and fully participate in the governance process in a free and voluntary manner to achieve consensus on a specific goal and outcome, and play their respective roles.” Gil-Garcia et al. (20) argued that in the future, cross-organizational cooperation and information sharing will be increasingly needed to address complex public problems. The information needs of different cooperating organizations may vary significantly in terms of completeness and timeliness, and these differences require a clear definition of the roles and responsibilities of all parties involved in the cooperation and information sharing processes between government organizations. In this process, the participating parties need to consider both government and non-government organizations. Kapucu et al. (21) studied the relationships between governments and organizations in collaborative emergency management and suggested that emergency management work requires the integration of the organizational culture, structure, and processes of various stakeholders. Additionally, effective use of resources by the collaborative network of stakeholders is required to meet the high expectations of the public and stakeholders for emergency and disaster management. Kim (22) suggested that information collaboration based on effective collaboration between institutions and members can help organizations achieve common goals, such as efficiently responding to public health emergencies.

In summary, research on information collaboration in public health emergencies has mostly focused on its fixed features, static structures, and optimization paths, with limited attention given to its dynamic characteristics from an evolutionary perspective. To address this gap, the present study employs the theory of complex adaptive systems and introduces a time-slicing approach to segment information collaboration in public health emergencies into distinct stages. Social network analysis methods are then utilized to examine the information collaboration process at each phase, uncover its evolutionary patterns, and pinpoint the evolution cycle of information collaboration in such emergencies. The ultimate goal of this study is to serve as a reference for enhancing emergency strategies for public health emergencies in China.

## 3. Analysis of information coordination in public health emergencies

### 3.1. Definition of information coordination in public health emergencies

In the era of informatization, the role of information coordination in public health emergencies has become indispensable, as the urgency of timely information sharing and data analysis is paramount for event prevention, control, and treatment. To fully appreciate the intricacies of information coordination, it is imperative to clarify certain terminologies, especially the term ‘preparedness’ which holds significant weight in discussions about public health emergencies. ‘Preparedness’ in the context of public health refers to the systematic and continuous process of planning and implementing measures to prevent, respond to, and recover from potential public health threats and emergencies. It encompasses a wide range of activities, from risk assessments and capacity building to the establishment of communication channels and the formation of response strategies. A key facet of preparedness is the ability to anticipate potential crises and have protocols in place that ensure quick and effective action. Intertwined with the notion of preparedness is information coordination. Information coordination is not solely about the dissemination and transmission of data. It emphasizes enhancing the utilization efficiency of the shared information through intricate interactions among various information entities and between these entities and their environment (23). This process involves not just augmenting the collaboration among these entities through efficient transmission methods but also focusing on extracting the utmost value from the shared information. The intricacies of information coordination in public health emergencies encompass a multitude of information entities. The modes and types of coordination among these entities can be vast and multifaceted, highlighting the need for meticulous information sharing mechanisms to ensure optimal preparedness (24). Given the inherent unpredictability of public health crises, such coordination becomes a linchpin in ensuring that accurate and relevant information is disseminated to appropriate entities promptly, facilitating swift and pertinent responses.

### 3.2. Characteristics of information coordination in public health emergencies

This study uses the theory of complex adaptive systems to determine whether the information coordination process in public health emergencies can undergo self-evolution. According to the theory of complex adaptive systems, entities in a system have learning capabilities. They continually “learn” and “accumulate experience” in their interactions with the environment and other entities, which is known as adaptability (25). Thus, as individual and environmental adaptive behaviors occur, the system undergoes self-evolution (26). If the information coordination in public health emergencies belongs to a complex adaptive system, it will undergo evolution. The following section analyzes whether the development of information coordination in public health emergencies exhibits the five basic characteristics of aggregation, nonlinearity, flow, diversity, and internal mechanisms to further determine whether it belongs to a complex adaptive system.

#### 3.2.1. Diversity

Diversity is an inherent characteristic of complex adaptive systems that refers to the continuous differentiation among individuals during the adaptation process, leading to a diverse array of responses within the system (27). In the context of information coordination in public health emergencies, diversity manifests as variations in chosen methods of coordination by different entities across various regions, and even temporal variations within the same region. This diversity is pivotal for public health emergency preparedness and response as it mirrors the heterogeneity of such crises. Diverse methods of information coordination not only reflect the unique characteristics and capacities of the involved entities, but they also accommodate the varying nature of emergencies across different contexts. For instance, the response to an emerging infectious disease might require different coordination strategies compared to a chronic public health issue. Even within the same public health emergency, the stages of the crisis may call for different approaches, such as rapid response in the initial phase and recovery efforts in the aftermath. Moreover, from a gender perspective, it is worth noting that diversity can also denote differential impacts of public health emergencies on different genders. Men and women often have different roles and responsibilities in their communities, which can lead to variations in their exposures and vulnerabilities during public health crises. As such, recognizing and incorporating this gender-based diversity in information coordination can contribute to more equitable and effective emergency responses. By identifying and respecting these diverse facets of information coordination, decision-makers can devise more tailored strategies, improving the resilience and efficacy of public health emergency responses. Therefore, diversity is not just an observable phenomenon, but a necessary characteristic that warrants careful consideration in the information coordination process of public health emergencies.

#### 3.2.2. Aggregation

Aggregation, as a key characteristic of complex adaptive systems, signifies the formation of larger aggregates through “adhesion” between individuals during the adaptation process (28). In public health emergencies, this translates into how various entities (like the government, public, social organizations, market, and healthcare institutions) come together to form a unified front for coordinated response efforts. The aggregation of these entities is more than a mere summation of their individual parts; it creates a synergy that allows for a more comprehensive, coordinated response to public health emergencies. It enables a multi-faceted approach to information coordination, where each entity brings its unique perspective and resources to the table, enhancing the richness and reach of the shared information. In the context of preparedness, the process of aggregation promotes a unity of effort and purpose, fostering collective decision-making and responsibility sharing. This collaborative approach allows for the harnessing of a wider range of resources and knowledge, paving the way for more robust responses to public health emergencies. Moreover, aggregation in information coordination is not only about the convergence of entities but also the convergence of different types of information. This could include epidemiological data, health system capacities, social determinants of health, and local community insights, among others. By aggregating diverse data sources, a more holistic view of the emergency can be achieved, supporting evidence-informed decision-making. Therefore, in the context of information coordination during public health emergencies, aggregation is not merely an inherent attribute, but a crucial operational principle that promotes effective response coordination. It underscores the need for broad-based collaborations, fostering a synergy that is critical for comprehensive and effective emergency management.

#### 3.2.3. Flow

Flow is a crucial feature of complex adaptive systems, referring to the exchange of matter, energy, and information between individuals and their environment (29). In public health emergencies, the concept of flow can be understood as the circulation of information amongst different entities and stakeholders. The flow of information is not a unidirectional transmission, but a dynamic process of exchange, interpretation, and feedback. It plays an indispensable role in the timely and effective response to public health emergencies, ensuring that crucial data and insights reach the right people at the right time. The importance of flow in preparedness cannot be overstated. It directly impacts the speed of response, the accuracy of actions, and ultimately, the effectiveness of interventions. For instance, quick and accurate flow of epidemiological data can expedite the detection and understanding of a novel disease, leading to timely and targeted interventions. However, information flow in public health emergencies is also fraught with challenges, including information overload, misinformation, and disparities in information access. These issues underscore the need for well-coordinated mechanisms to manage the flow of information, including verification systems to ensure data accuracy, strategies to disseminate clear and consistent messages, and equitable information access strategies. Moreover, the concept of flow also points to the need for interoperable data systems that allow seamless sharing and integration of data across different platforms and entities. This ensures that information is not siloed within individual entities, but can be effectively used and built upon by others. In conclusion, the characteristic of flow in the information coordination process during public health emergencies is not only about the exchange of information but also about the management of that exchange. It emphasizes the need for well-coordinated mechanisms to facilitate effective and equitable information flow, underscoring the interconnectedness and interdependence of all entities involved in emergency response.

#### 3.2.4. Non-linearity

Non-linearity is a fundamental characteristic of complex adaptive systems, pointing out that the interactions between entities in the system do not follow simple linear relationships but are non-linear (30). This is particularly evident in the context of public health emergencies, where multiple entities with different roles and responsibilities interact and coordinate in a non-linear and dynamic manner. In public health emergencies, the success of information coordination does not directly correlate with the improvement of a single entity’s ability. For instance, focusing only on enhancing the capability of medical departments in assimilating and transmitting information may not necessarily lead to an improved overall information coordination efficiency. This is because the efficiency of information coordination relies not just on the capacity of a single entity, but on the collective capabilities of all entities involved, including communities, markets, government institutions, and the public. The concept of non-linearity also illuminates the interconnected nature of the entities in the system. It emphasizes that all entities in the system are interdependent and that changes in one entity can have ripple effects on others. This underscores the need for a comprehensive and holistic approach in improving information coordination. Strategies should not just target individual entities but should consider the system as a whole, ensuring that enhancements in one area support and are supported by enhancements in other areas. In conclusion, the non-linear nature of information coordination in public health emergencies highlights the importance of a systemic approach in enhancing information coordination. It draws attention to the interconnectedness and interdependence of all entities involved, reinforcing the need for collective capabilities and cooperative efforts in managing information in public health emergencies.

#### 3.2.5. Internal model

The internal model mechanism of complex adaptive systems refers to the fixed models or protocols for problem-solving that entities adhere to when they encounter new information or challenges within the system (31). The capacity to develop, adopt, and modify internal models in response to new information is crucial in the context of information coordination during public health emergencies. Take, for example, the city of Wuhan after suffering a major blow from the 2020 epidemic. The city summarized its experiences, strengthened information infrastructure, and created the “Wuhan Health Code.” This was an innovative solution that ensured the interconnection and interoperability of information across multiple sectors, thereby improving information transmission in prevention and control efforts. It served as an internal model that effectively optimized the overall information coordination mechanism in the face of an unprecedented public health crisis. The internal model mechanism underlines the importance of learning and adaptability in public health emergencies. Entities need to continuously learn from their experiences, adapt their models and strategies, and be prepared to devise new models in response to changing circumstances. This adaptability not only improves the efficiency of information coordination but also enhances the resilience of the system to future public health emergencies. In summary, the internal model mechanism characteristic of complex adaptive systems accentuates the need for continuous learning, adaptability, and innovative problem-solving in information coordination during public health emergencies. It exemplifies the role of effective internal models in enhancing the efficiency of information coordination and the overall resilience of the system.

Based on the above analysis, information coordination in public health emergencies belongs to complex adaptive systems. Therefore, the information coordination process of public health emergencies exhibits adaptive characteristics of complex adaptive systems and can undergo autonomous evolution. On this basis, the following section introduces time slicing, transforming the dynamic changes of the information coordination process into planar slices, and further analyzing its evolutionary process.

## 4. Materials and methods

### 4.1. Research tool

In this study, cohesive subgroups in social network analysis are selected as the analytical tool to investigate the information collaboration and evolution process. Cohesive subgroup analysis is a type of social structure research that examines the existing or potential relationship patterns between social actors. These relationship patterns can take various forms, including dyadic relationships, triadic relationships, and subgroup-level relationships. A cohesive subgroup in a network is characterized by the following features: (1) strong ties among nodes within the subgroup and weak ties between subgroups, and (2) a subgroup is a set of individuals in a network that are tightly connected within the subgroup but loosely connected outside the subgroup.

As a result of the impact of public health emergencies on transportation, education, healthcare, production, and other fields, information collaboration during such emergencies is also extremely complex, with significant differences in the ways actors communicate, the frequency of communication, and the content of information exchanged. Analyzing the cohesive subgroups in the information collaboration network during public health emergencies can help identify closely connected actors in the collaboration process, thereby clarifying the distribution of rights and responsibilities among different actors and the structure of the information collaboration network. This is of significant importance for analyzing complex networks.

### 4.2. Selection of research objectives

In January 2020, the COVID-19 outbreak occurred in China, posing a significant threat to people’s lives, health, and property. Various sectors of society have participated in the prevention and control of the epidemic in different ways. Due to the complex relationships between the organizations and individuals involved in epidemic prevention and control, there has been a large amount of related information, in various forms and with different transmission paths. Therefore, this study chooses various types of information generated by the actors involved in the COVID-19 epidemic as research objects.

### 4.3. Data sources

Data for this research was predominantly informed by the provisions of the “Emergency Response Law of the People’s Republic of China” and the “National Management Specifications for Reporting Relevant Information on Public Health Emergencies (Trial).” These statutes identify the key information producers during public health emergencies as the government, public, social organizations, the market, and medical institutions. Hence, the study classifies the actors engaged in information collaboration during public health emergencies into these five categories. This classification necessitates sourcing data from the official websites of various organizations that disseminate epidemic-related information. Given the urgent nature of public health emergencies, these sites are acknowledged as reliable and timely data sources. The rapid evolution of situations such as the COVID-19 pandemic underscores the importance of immediate and accurate sources, which considerably influenced the decision to utilize these platforms for this study. To facilitate data collection, a set of search terms, aligned with the identified actor categories, was established. These terms guided the systematic exploration and collection of information from the relevant websites, as detailed in Table 1.

**Table 1:** Framework of user health information requirements.

Information entities	Information entities
Government	National Health Commission, Centers for Disease Control and Prevention, Ministry of Finance, Ministry of Industry and Information Technology, Ministry of Civil Affairs, State Council, National Development and Reform Commission, Life Necessities Security Group, Ministry of Agriculture and Rural Affairs, Ministry of Commerce, Ministry of Education, China Banking and Insurance Regulatory Commission, Medical Administration Bureau, etc.
Market	Operating Stores, Online Course Platforms, Farmer’s Markets, Seafood Markets, China Railway Group Limited, Pharmaceutical Companies, Supply and Marketing Cooperatives, Recreational Agriculture, Rural Tourism Operators, Enterprises, etc.
Social Organizations	World Health Organization, Chinese Academy of Engineering, schools, Chinese Academy of Medical Sciences, Chinese Academy of Sciences, Academy of Military Medical Sciences, Xinhua News Agency, People’s Daily Online, Chao Wen Tian Xia, People’s Daily, Red Cross Society of Hubei Province, Hubei Charity Federation, Hubei Youth Development Foundation, etc.
Healthcare	Medical Institutions, Medical and Health Institutions, Cabin Hospitals, Fever Clinics, Designated Medical Institutions, Provincial Hospitals, Community Health Service Centers, Designated Medical Points, etc.
General Public	Village Committees, Communities, Neighborhood Committees, Citizens, Village Self-Governing Organizations, etc.

While this approach primarily confines the study’s data sources to governmental websites, it nonetheless provides a comprehensive overview of collaborative processes during public health emergencies. Despite the limitations in the diversity of data sources, this methodology ensures reliability, timeliness, and direct relevance to the research question. As such, it holds considerable value in comprehending the evolution of information collaboration in such critical contexts.

**Figure 1:**
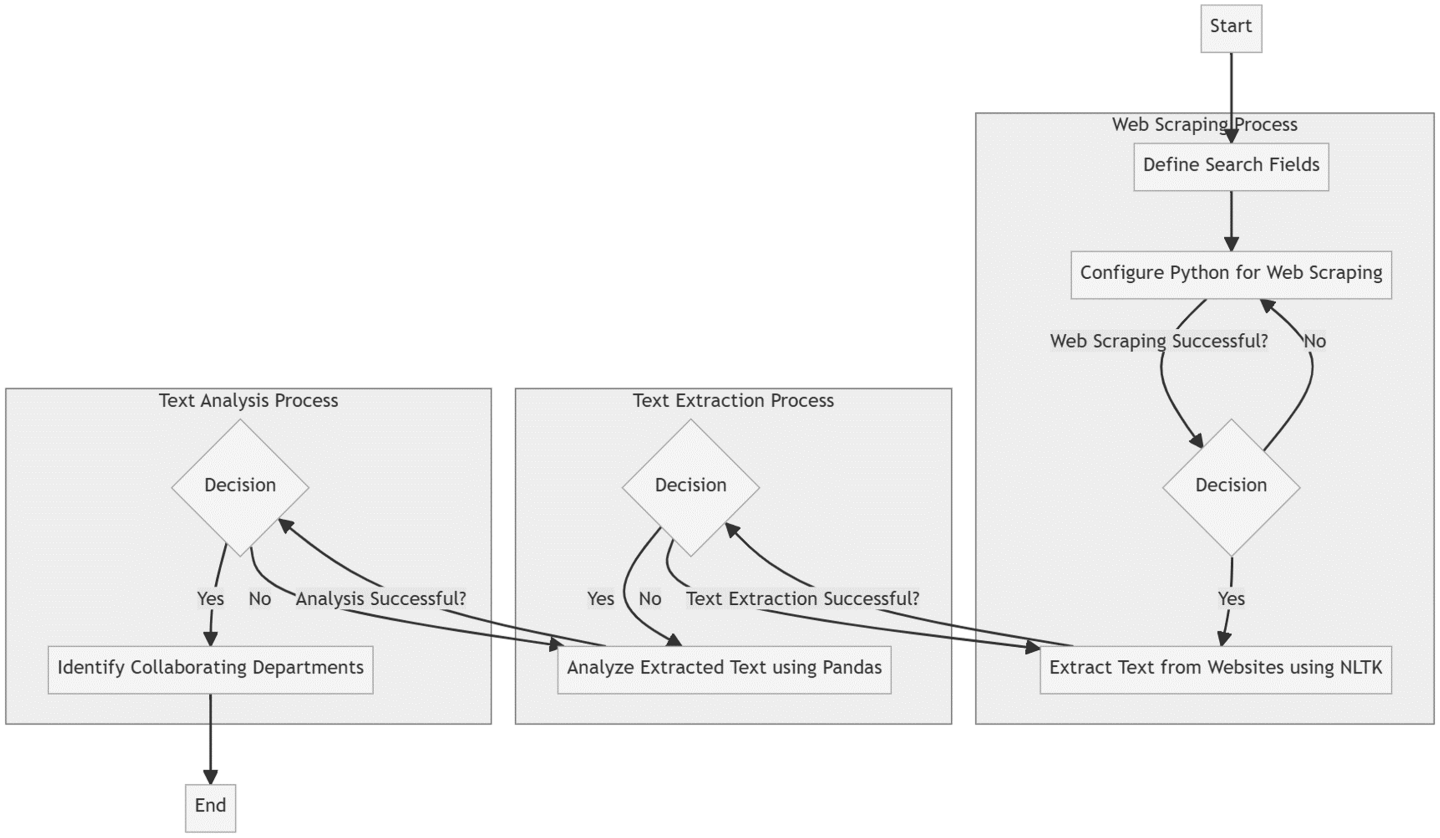
Flowchart illustrating the methodological steps used for web scraping, text extraction, and text analysis in the study.

In this study, Python software was employed to collect daily epidemic-related text data from the official websites of various ministries, which included the National Health Commission, the Chinese Government, the Ministry of Education, the Ministry of Transport, the Ministry of Finance, the Ministry of Public Security, and the Ministry of Civil Affairs. The data collection spanned from January 11, 2020, to April 15, 2020. Specific Python libraries, including Beautiful Soup for web scraping, Pandas for data manipulation and analysis, and NLTK for natural language processing, were employed for the task. The selection of these libraries was based on their proficiency in handling and processing web data and text analysis, respectively. Subsequently, search fields within the sites were established to optimize data collection. The study additionally involved the extraction of publishing agencies and the departments referred to in the text. When two departments co-published a text or were mentioned within the same text, they were deemed to be involved in the same collaboration. To enhance comprehension of the study’s methodology, a flowchart (Figure 1) detailing each step of the data collection and analysis process has been included.

### 4.4. Time slicing selection

This study introduced time slicing to explain the temporal evolution cycle of information collaboration in the epidemic. On January 11, 2020, the National Health Commission officially reported the outbreak of unknown pneumonia in Wuhan, marking the beginning of the COVID-19 epidemic. However, it had not yet developed into a major public health emergency threatening the whole country. On April 15, 2020, the epidemic in Hubei, which was the first area to experience a large-scale outbreak, was under basic control with zero new confirmed cases of COVID-19 and no imported cases. Based on these objective facts, this study focused on the temporal variation patterns of information collaboration related to the epidemic from January 11 to April 15, 2020.

To determine the number of time slices and the time span, this study analyzed three indicators: the number of departments, network density, and the average path length of the network. The number of departments represents the number of departments involved in the information collaboration network during a certain period. The overall network density represents the degree of closeness among the departments participating in the collaboration. The higher the network density, the closer the connection between the departments. The formula for calculating network density is $D_{i}=\frac{N}{M\left(M-1\right)}$, where *M* represents the number of nodes, and *N* represents the actual number of links. The average path length of the network represents the average collaboration distance between any two departments. The formula for calculating the average path length is $L=\frac{1}{C_{N}^{2}}\sum_{1\leq i\leq j\leq N}d_{ij}$, where *N* represents the number of departments, and *d_ij_* represents the collaboration distance between department *i* and department *j*. The calculation results are shown in Table 2.

**Table 2:** Analysis of information collaboration network.

Date	Number of Departments	Network Density	Average Path Length
1.11–1.17	6	1	1
1.18–1.25	14	0.446	1.619
1.26–2.1	22	0.548	1.452
2.2–2.8	34	0.401	1.615
2.9–2.15	38	0.45	1.55
2.16–2.22	41	0.537	1.463
2.23–2.29	32	0.46	1.54
3.1–3.7	29	0.426	1.574
3.8–3.14	24	0.408	1.391
3.15–3.21	38	0.489	1.511
3.22–3.28	34	0.466	1.534
3.29–4.4	26	0.489	1.511
4.5–4.11	27	0.499	1.501

The analysis results indicate that the number of departments showed a steady increasing trend in the early stage, with the first significant increase occurring on February 1st, when the number of participating departments reached 34. Subsequently, there were small fluctuations in the number of departments. On February 23rd, the number of departments showed a sharp decrease, twice the magnitude of the previous decline, but gradually recovered to a steady downward trend. On March 15th, the number of departments increased sharply again, followed by a continuous downward trend, with a large decrease occurring on March 29th.

The network density was initially high, reaching 0.548. Around February 1st, the network density decreased for the first time, dropping to 0.401, followed by a steady upward trend until around February 22nd, reaching a maximum of 0.537. On February 23rd, the network density showed a significant decrease to 0.46, and continued to decline until around March 15th. Then, the network density increased slightly and showed small fluctuations between March 15th and March 28th.

The average path length was initially high but showed a decreasing trend. There was a significant increase in the average path length around February 2nd, followed by a downward trend until around February 22nd when it reached 1.463. Then, it increased to 1.54 around February 24th and showed small fluctuations within a narrow range. Around March 15th, the average path length experienced a significant fluctuation, increasing from 1.391 to 1.511, and then continued to show small fluctuations around this value.

In summary, the information collaboration network experienced significant fluctuations on February 1st, February 22nd, March 14th, and March 28th, indicating significant changes in network status. Therefore, this paper divides the COVID-19 process into five time slices based on the dates of significant fluctuations: T1: January 11th – February 1st, T2: February 2nd – February 22nd, T3: February 23rd – March 14th, T4: March 15th – March 28th, T5: March 29th – April 11th.

## 5. Results

This article uses Python software to collect the occurrence frequency of each search field and the frequency of their associations based on the search fields. If two organizations participate in the same prevention and control measure, it is regarded as a collaborative effort, and the collected data is used as the basis for constructing the information collaboration network. The collected data is then processed by normalization. First, invalid fields are removed, and then duplicate fields in the content are manually filtered and irrelevant fields are cleared. The fields are then classified according to different time periods to obtain the information collaboration frequency for T1-T5. Finally, the corresponding information collaboration network graphs for T1-T5 are generated.

### 5.1. Time period T1

**Figure 2:**
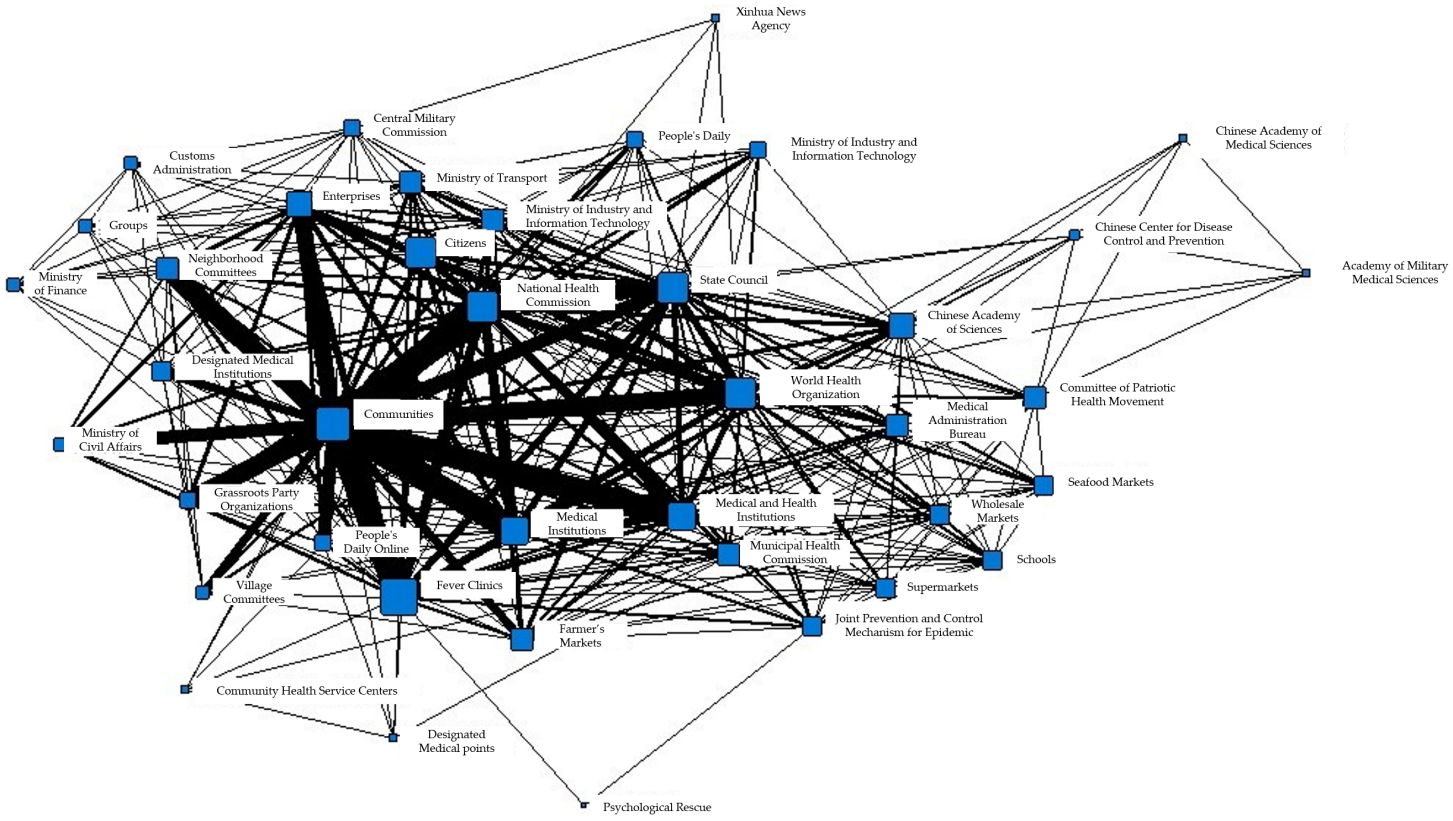
Collaborative network of epidemic information in T1 period.

Based on the data collected from the National Health Commission’s official website, the total number of collaborations between information subjects during T1 is 9,478, involving 40 information subjects. Among them, the community and fever clinics have the highest number of collaborations in the same prevention and control measures, with a total of 322 times. In addition, the community and medical institutions have the second-highest number of collaborative efforts, with a total of 318 times. The specific information collaboration network is shown in Figure 2.

Subsequently, the concordance subgroup analysis algorithm in the Ucinet software was employed to analyze the overall collaborative network structure, resulting in the identification of eight subgroups as shown in Table 3.

**Table 3:** Classification of cohesive subgroups in T1 period.

Cohesive subgroup	Search terms
1	Committee of Patriotic Health Movement, National Health Commission, Medical Administration Bureau, Joint Prevention and Control Mechanism for Epidemic, Psychological Rescue, Seafood Markets, Wholesale Markets, Farmers’ Markets, Schools, Chinese Academy of Sciences (Collaboration on Disinfection Action Information)
2	World Health Organization, Medical Institutions, State Council, National Health Commission (International Exchange Information Collaboration)
3	Military Medical Institute of Academy of Military Science, Chinese Academy of Medical Sciences (Virus Research Information Collaboration)
4	Citizens, Communities, Fever Clinics, People’s Daily (Collaboration on Disease Treatment Information)
5	People’s Daily Online, Grassroots Party Organizations, Ministry of Civil Affairs, Village Committees (Collaboration on Health Monitoring Information)
6	Chinese Center for Disease Control and Prevention
7	Customs Administration, Ministry of Finance, Enterprises, Designated Medical Institutions, Ministry of Transport, Central Military Commission, Ministry of Industry and Information Technology, Neighborhood Committees
8	Community Health Service Centers, Designated Medical Institutions

**Figure 3:**
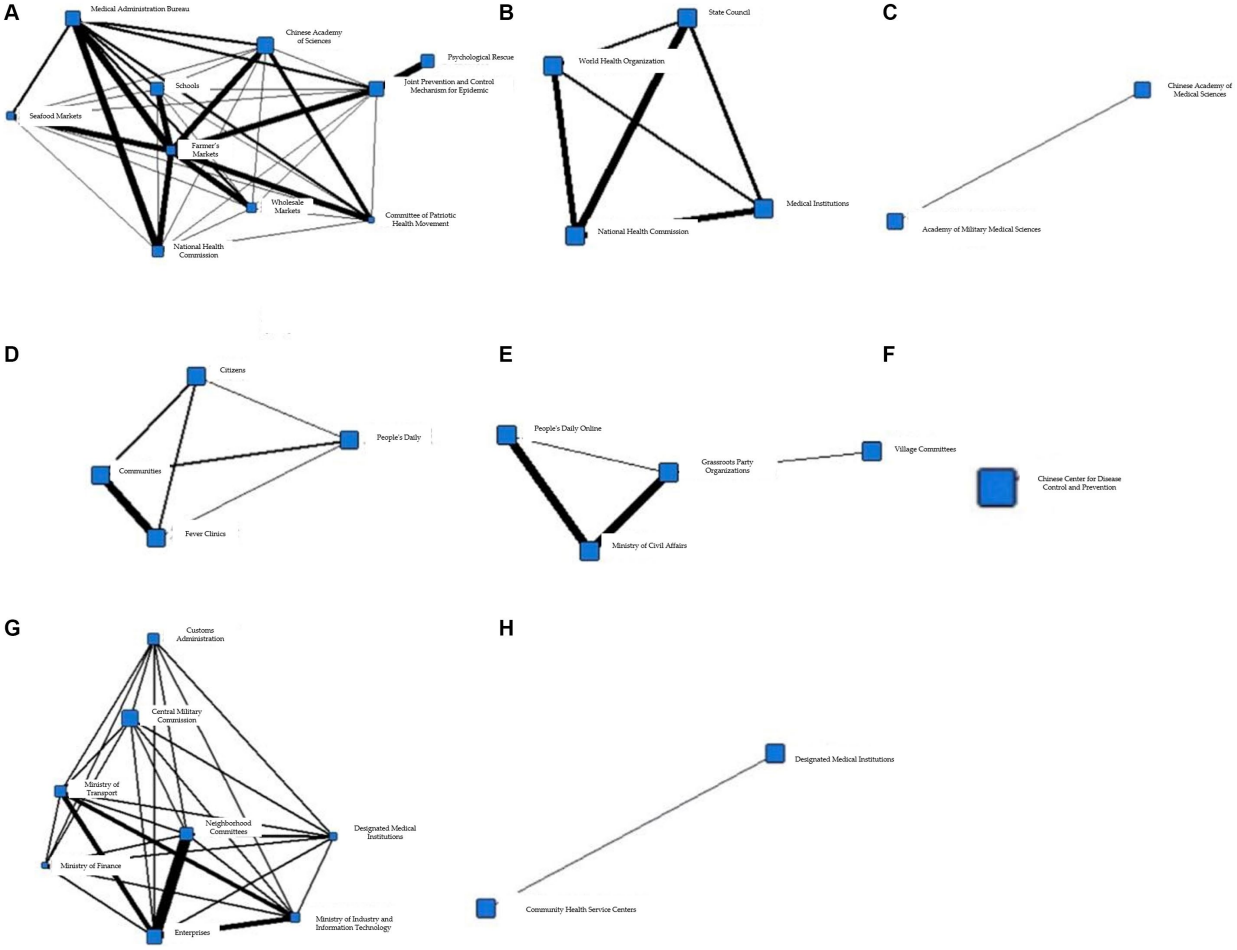
**(A)** Coherent subgroup 1 information collaboration network diagram during T1 period. **(B)** Coherent subgroup 2 information collaboration network diagram during T1 period. **(C)** Coherent subgroup 3 information collaboration network diagram during T1 period. **(D)** Coherent subgroup 4 information collaboration network diagram during T1 period. **(E)** Coherent subgroup 5 information collaboration network diagram during T1 period. **(F)** Coherent subgroup 6 information collaboration network diagram during T1 period. **(G)** Coherent subgroup 7 information collaboration network diagram during T1 period. **(H)** Coherent subgroup 8 information collaboration network diagram during T1 period.

To better illustrate the information coordination within different subgroups during the T1 period, Gephi software was used to generate the following network diagram. Figures 3A–H show the interactions among various departments within 8 cohesive subgroups:

Figure 3A demonstrates the information coordination within Cohesive Subgroup 1, which includes government, market, and social organizations as information nodes. Government information nodes, represented by the Committee of Patriotic Health Movement and the Municipal Construction Committee, are closely linked to market information nodes, represented by the Agricultural Products Market, while also maintaining close relationships with social organizations, represented by schools. Based on relevant reports associated with these fields, it is clear that these departments had obvious information coordination in environmental disinfection tasks. Figure 3B shows the information coordination within Cohesive Subgroup 2, which includes medical, government, and social organizations as information nodes. Social organizations, represented by the World Health Organization, have close information exchanges with domestic medical institutions and the State Council for international information exchanges, according to the corresponding reports. The coordination among these nodes aims to achieve international information exchange. Figure 3C shows the information coordination within Cohesive Subgroup 3, where all nodes belong to social organizations, mainly including various research institutes with virus research as the primary goal. Cohesive Subgroup 4 (Figure 3D) mainly includes the public and medical information nodes. This subgroup includes citizens and fever clinics, and according to relevant reports, their main information coordination content is related to diagnosis and treatment of cases. Figure 3E demonstrates the information coordination within Cohesive Subgroup 5, which mainly includes public and government information nodes. The primary purpose of their information coordination is to monitor public health and promote disease prevention. Cohesive Subgroup 6 (Figure 3F) includes only one information node, the Centers for Disease Control and Prevention, and according to relevant reports, the CDC has information coordination with other nodes, and the frequency is similar. Therefore, it can be inferred that there is a great demand for the CDC’s information processing ability during a public health emergency in the T1 phase. Figure 3G shows the information coordination within Cohesive Subgroup 7, which mainly includes medical, market, and government information nodes, with the main purpose of coordinating the movement of people. As the novel coronavirus is transmitted through droplets, reducing gatherings and avoiding contact is crucial for stopping the spread of the virus. Figure 3H demonstrates the information coordination within Cohesive Subgroup 8, which includes only medical information nodes and represents information coordination between grassroots hospitals and higher-level hospitals.

Based on the above analysis, it can be concluded that there was no overall information coordination during the T1 period, but multiple small-scale information coordination networks were formed. In addition, the properties of information nodes within each cohesive subgroup were relatively homogeneous. Cohesive Subgroup 1 had the largest number of nodes and the most frequent coordination, making it the main component of the information coordination network during this period. According to the corresponding news reports for Subgroup 1, the focus of epidemic prevention and control during this phase was on disinfection of key locations and protection of special populations, with overall coordination centered around the market and networking at different levels. Based on these coordination situations and related reports, it can be inferred that there was no comprehensive participation in information coordination by the whole society during this stage, and different information nodes played their own roles, with information coordination involving relatively narrow areas. The epidemic did not pose a serious threat to society at that time.

### 5.2. Time period T2

**Figure 4:**
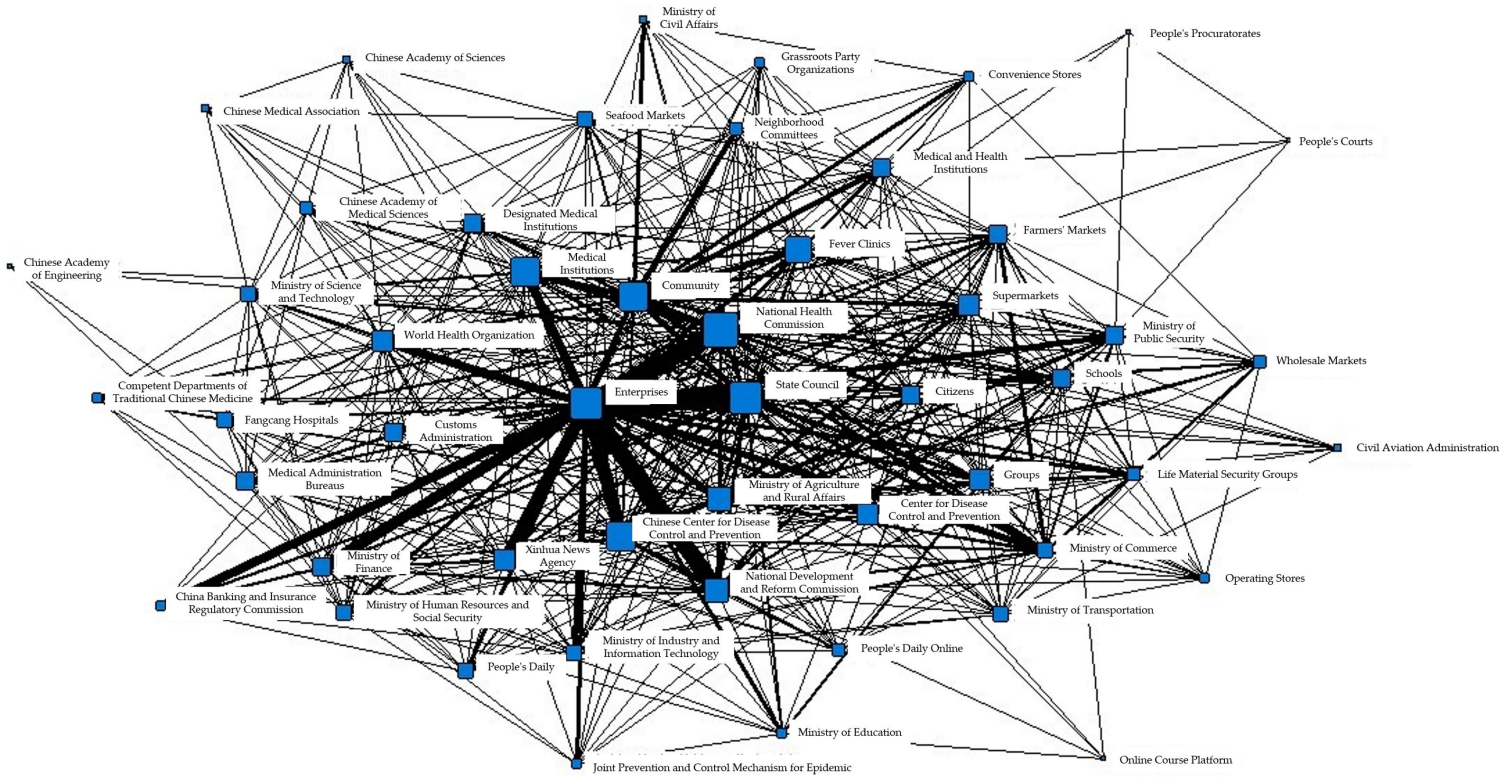
Collaborative network of epidemic information in T2 period.

The total number of information collaborations in T2 was 39,540, involving 51 search fields. Among them, the closest associations were observed between enterprises and the State Council, with a collaboration count of 1,516. In addition, the collaboration counts between enterprises and the National Health Commission were 1,260, while those between enterprises and the National Development and Reform Commission, as well as the Ministry of Industry and Information Technology, were both 1,256. The specific information collaboration network is shown in Figure 4.

The concordance subgroup analysis algorithm in the Ucinet software was used to analyze the overall information collaboration structure, and eight subgroups were identified as shown in Table 4.

**Table 4:** Classification of cohesive subgroups in T2 period.

Cohesive Subgroup	Search Terms
1	Convenience Stores, Grassroots Party Organizations, Chinese Center for Disease Control and Prevention, Medical and Health Institutions, Ministry of Civil Affairs, Citizens, Farmers’ Markets, Civil Aviation Administration, Neighborhood Committees, Online Course Platforms, Schools.
2	People’s Courts, People’s Procuratorates.
3	Wholesale Markets, Operating Stores, Ministry of Public Security, Supermarkets, Ministry of Transportation, Ministry of Commerce, Life Material Security Groups, Groups.
4	Ministry of Agriculture and Rural Affairs, Fever Clinics, State Council.
5	People’s Daily, Joint Prevention and Control Mechanism for Epidemic, People’s Daily Online, China Banking and Insurance Regulatory Commission, National Development and Reform Commission, Ministry of Industry and Information Technology.
6	Community, Medical Institutions, Designated Medical Institutions, Health Commissions, Chinese Center for Disease Control and Prevention, World Health Organization, Enterprises.
7	Ministry of Science and Technology, Seafood Markets, Chinese Academy of Medical Sciences, Chinese Medical Association, Chinese Academy of Sciences.
8	Fangcang Hospitals, Medical Administration Bureaus, Customs Administration, Chinese Academy of Engineering, Ministry of Human Resources and Social Security, Ministry of Industry and Information Technology, Ministry of Finance, Competent Departments of Traditional Chinese Medicine.

**Figure 5:**
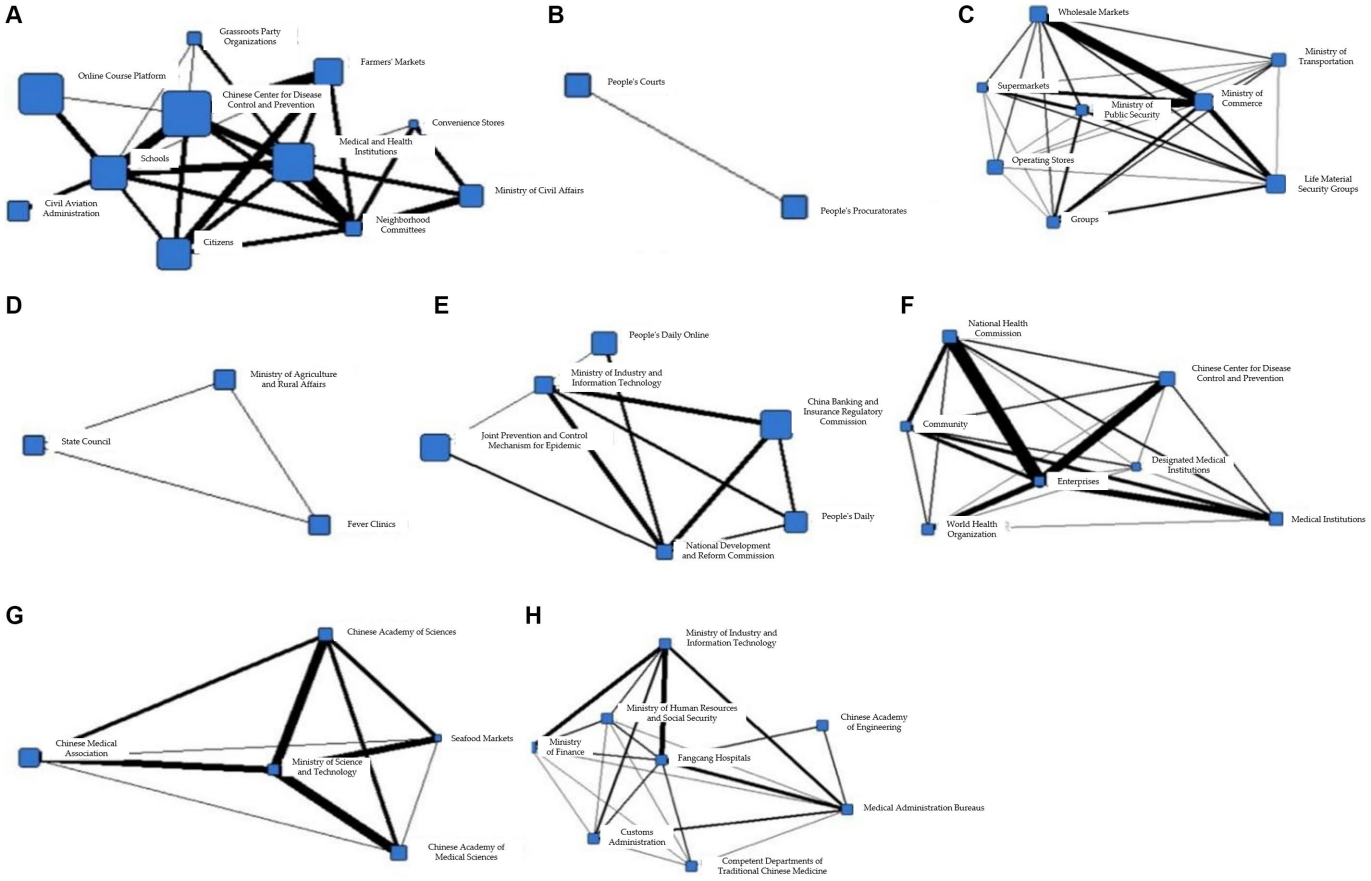
**(A)** Coherent subgroup 1 information collaboration network diagram during T2 period. **(B)** Coherent subgroup 2 information collaboration network diagram during T2 period. **(C)** Coherent subgroup 3 information collaboration network diagram during T2 period. **(D)** Coherent subgroup 4 information collaboration network diagram during T2 period. **(E)** Coherent subgroup 5 information collaboration network diagram during T2 period. **(F)** Coherent subgroup 6 information collaboration network diagram during T2 period. **(G)** Coherent subgroup 7 information collaboration network diagram during T2 period. **(H)** Coherent subgroup 8 information collaboration network diagram during T2 period.

To better illustrate the information collaboration among different information entities within each subgroup during the T2 period, the Gephi software was used to generate the following network diagrams. Figures 5A–H respectively show the interaction among various departments in the eight concordance subgroups.

Based on Figure 5A, it can be seen that the five main information entities in Cohesive Subgroup 1 are closely related. The government information entities represented by the Civil Aviation Administration, the Ministry of Civil Affairs, and the Centers for Disease Control and Prevention; the social organization information entities represented by schools, and the market information entities represented by agricultural markets are the most closely linked. Combining the relevant reports on the appearance of the fields, it can be inferred that the main purpose of information synergy in Cohesive Subgroup 1 is to block transmission channels, reduce population gatherings, and reduce the probability of virus transmission through patriotic hygiene campaigns.

Figure 5B shows that Cohesive Subgroup 2 only includes government information entities – the People’s Court and the People’s Procuratorate. By searching for corresponding reports, it can be known that the main purpose of synergy in this subgroup is to establish regulations to ensure the safety of medical personnel and maintain good medical order.

Figure 5C shows that Cohesive Subgroup 3 mainly includes government and market information entities. The Ministry of Commerce, wholesale markets, the Ministry of Public Security, and the living materials guarantee group under the epidemic prevention and control mechanism are the most closely linked. By searching for corresponding reports, it can be known that the information synergy task in this subgroup is to guarantee the production and transportation of daily necessities and medical protective equipment.

Figure 5D shows the information synergy situation in Cohesive Subgroup 4, which mainly involves government and medical information entities, including the Ministry of Agriculture and Rural Affairs, fever clinics, and the State Council. According to Figure 5D and corresponding reports, it can be known that the planting and animal husbandry bureaus under the Ministry of Agriculture and Rural Affairs participate most frequently, and the main purpose of information synergy in this subgroup is to ensure the normal spring plowing and stable production of important agricultural products.

Figure 5E shows that Cohesive Subgroup 5 mainly consists of information synergy within the government information entities. Based on relevant reports, it can be known that the main purpose of information synergy is to maintain financial security and market stability from a macro perspective, and ensure that epidemic prevention and control funds, employee salaries, and basic livelihoods are not affected.

Figure 5F shows the information synergy situation in Cohesive Subgroup 6, which includes government, public, market, and medical information entities. It can be observed from the figure that enterprises and other information entities are most frequently linked. According to corresponding reports, the main purpose of information synergy in this subgroup is to support resumption of work and ensure stable production line operation.

Figure 5G shows the information synergy situation in Cohesive Subgroup 7, which mainly includes social organization and market information entities, with various research institutions as the main social organization entities. Since this round of epidemic is believed to have originated from a seafood market in Wuhan, the seafood market inevitably became a key research object in the study of the virus. Combining relevant reports, it can be determined that the main purpose of information synergy in this subgroup is virus research.

Figure 5H shows the information synergy situation in Cohesive Subgroup 8, which includes government, medical, and social organization information entities. It can be seen from the figure that the Fangcang Hospital is at the center of information synergy. In the T2 stage, with the sudden increase in cases, Fangcang Hospital gradually became an important response measure. According to corresponding reports, the main purpose of information synergy in this subgroup is to ensure the normal operation of Fangcang Hospital.

Based on the above analysis, it can be seen that the complexity within each sub-cluster has increased during the T2 phase. The high degree of overlap in node attributes within different sub-clusters indicates that a single entity may participate in multiple tasks simultaneously (e.g., the government may participate in the information collaboration of sub-clusters a, b, c, d, e, h), resulting in information collaboration involving multiple sectors, such as legal, medical, economic, transportation, and education. As shown in the figure, the government departments are located at the core positions within sub-clusters a, c, and g, while enterprises and hospitals are at the core positions in sub-clusters f and h, respectively. This analysis suggests that during this period, the influence of the epidemic had gradually expanded to the social scope, and the government, enterprises, and hospitals played important roles in the overall information collaboration.

### 5.3. Time period T3

**Figure 6:**
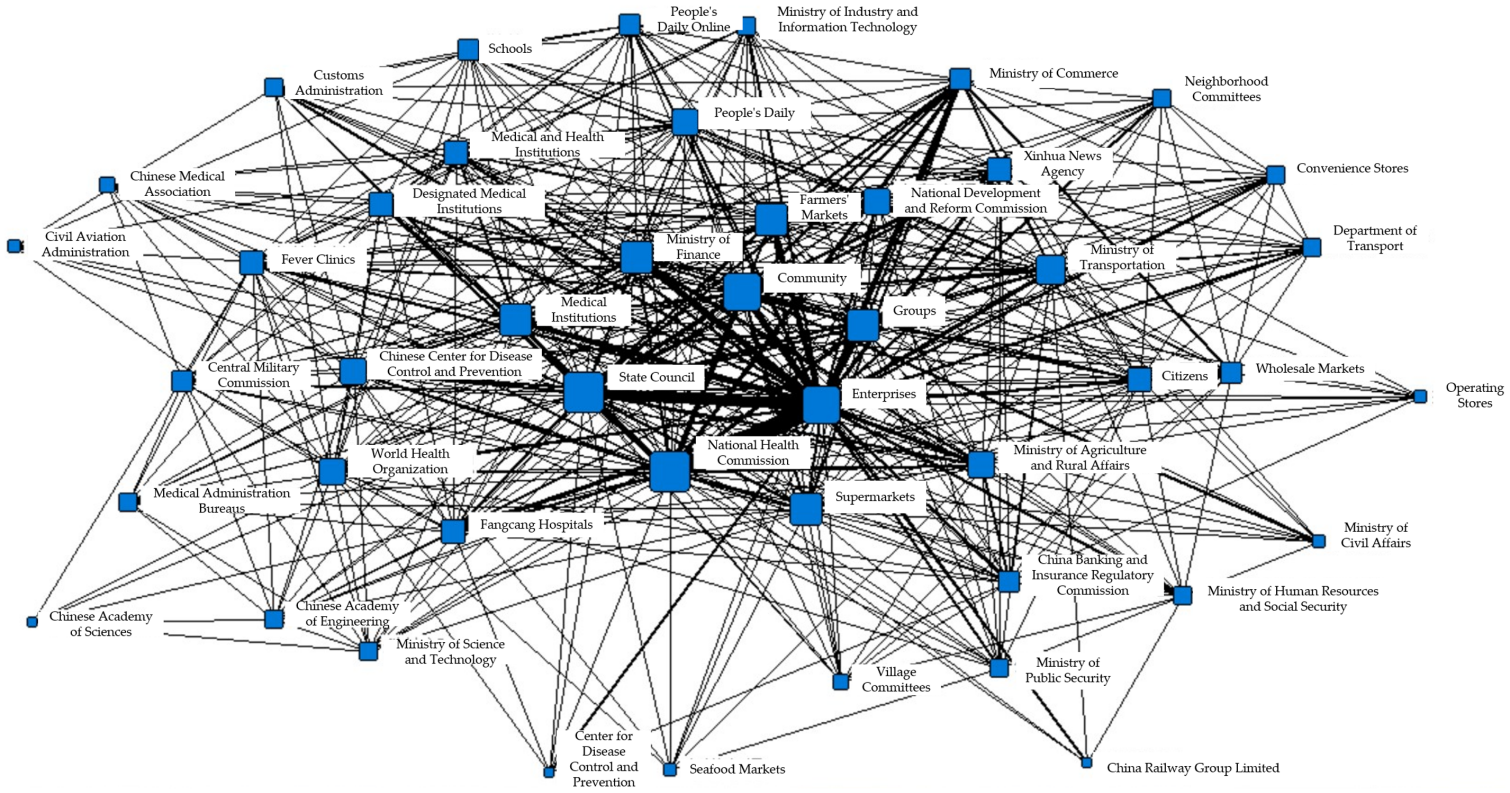
Collaborative network of epidemic information in T3 period.

The total number of collaborations in T3 was 28,829, with 46 search fields appearing in the above figure. The most closely related collaborations included 1,206 collaborations between enterprises and the State Council, and 1,918 collaborations between enterprises and the National Health Commission, as shown in Figure 6.

Furthermore, the concor analysis algorithm in the Ucinet software was used to analyze the overall information collaboration structure and generate the following eight cohesive subgroups, as shown in Table 5.

**Table 5:** Classification of cohesive subgroups in T3 period.

Cohesive Subgroup	Search Terms
1	Convenience Stores, Farmers’ Markets, Department of Transport, Wholesale Markets, Neighborhood Committees, National Development and Reform Commission, People’s Daily, Ministry of Transport, Ministry of Commerce, Citizens
2	Communities, Enterprises, Supermarkets
3	China Banking and Insurance Regulatory Commission, Village Committees, Ministry of Agriculture and Rural Affairs, Ministry of Industry and Information Technology, Ministry of Human Resources and Social Security
4	Ministry of Civil Affairs, Ministry of Public Security, Seafood Markets
5	Designated Medical Institutions, People’s Daily, Ministry of Industry and Information Technology, Medical Institutions, Ministry of Finance, Fever Clinics
6	Customs Administration, Civil Aviation Administration, Schools
7	World Health Organization, Central Military Commission, Fangcang Hospitals, Center for Disease Control and Prevention, Health Commission, Chinese Center for Disease Control and Prevention, Chinese Academy of Engineering, Ministry of Science and Technology, Chinese Academy of Sciences
8	National Health Commission, State Council.

**Figure 7:**
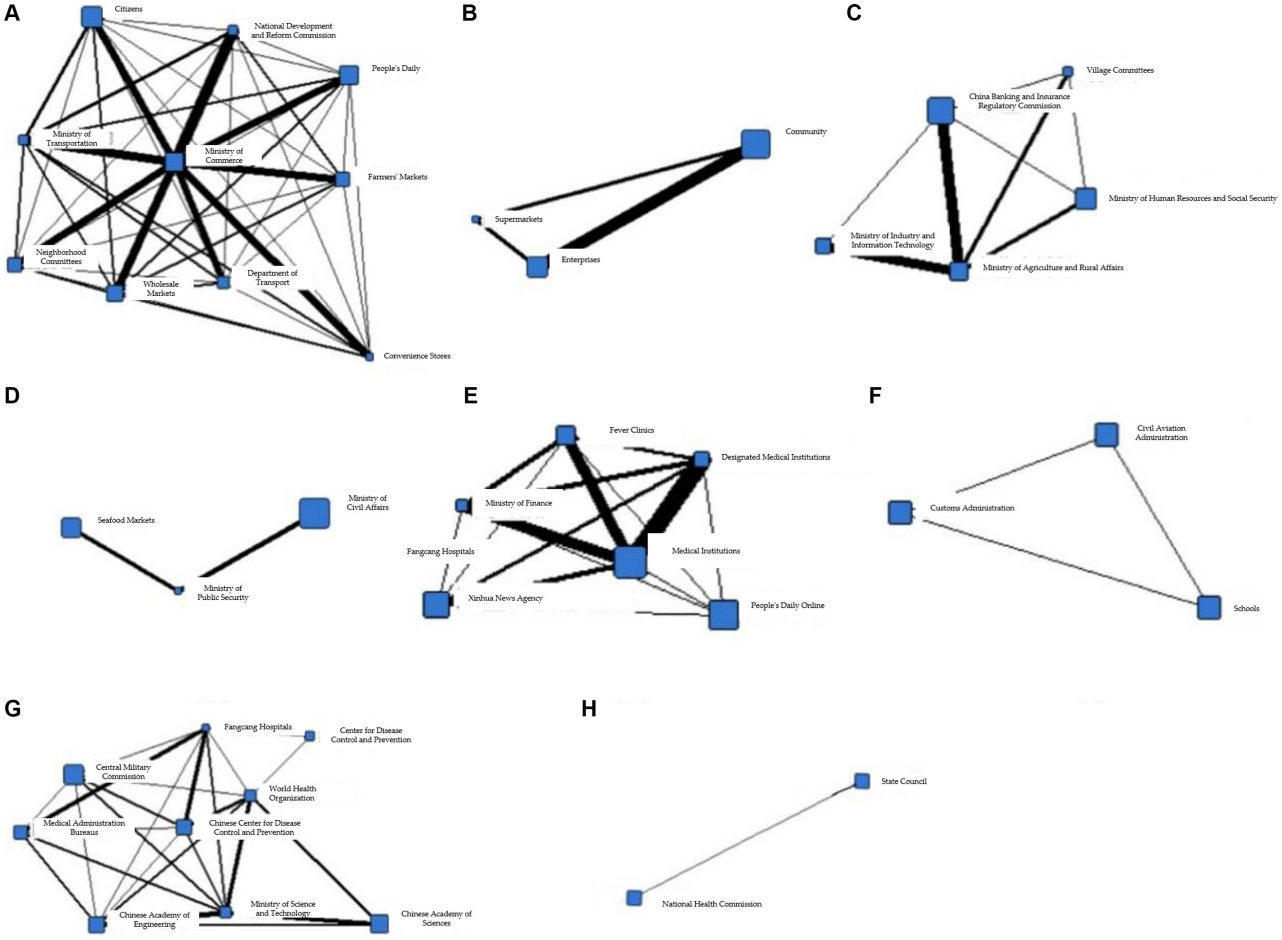
**(A)** Coherent subgroup 1 information collaboration network diagram during T3 period. **(B)** Coherent subgroup 2 information collaboration network diagram during T3 period. **(C)** Coherent subgroup 3 information collaboration network diagram during T3 period. **(D)** Coherent subgroup 4 information collaboration network diagram during T3 period. **(E)** Coherent subgroup 5 information collaboration network diagram during T3 period. **(F)** Coherent subgroup 6 information collaboration network diagram during T3 period. **(G)** Coherent subgroup 7 information collaboration network diagram during T3 period. **(H)** Coherent subgroup 8 information collaboration network diagram during T3 period.

The cohesive subgroup network generated during the T3 phase is shown in Figure 7, with a-h demonstrating the information collaboration situation of each department in the eight cohesive subgroups, respectively.

From Figure 7A, it can be seen that the information synergy within Cohesion Subgroup 1 includes information subjects such as government, market, the public, and social organizations. Based on corresponding reports, the main purpose of information synergy in this subgroup is to ensure the transportation and distribution of daily necessities. Figure 7B shows the information synergy situation within Cohesion Subgroup 2, which includes information subjects such as the public and the market. As can be seen from the figure, information synergy has been generated due to the resumption of work and production and the consequent movement of people. Cohesion Subgroup 3, as shown in Figure 7C, mainly involves information synergy between government and the public, with the main purpose being to maintain the supply of necessities such as vegetables, meat, eggs, and milk, as well as to stabilize market prices. Figure 7D shows the information synergy involved in Cohesion Subgroup 4, which is between the market, the Ministry of Public Security, and the Ministry of Civil Affairs. According to corresponding reports, the main purpose of information synergy in this subgroup is to crack down on illegal wildlife markets and trade, and cut off the transmission of the epidemic virus at the source. Figure 7E shows the information synergy within Cohesion Subgroup 5, which includes government and medical information subjects. According to the figure, the Ministry of Industry and Information Technology is at the center of information synergy, and based on reports, the main purpose of information synergy is to support the resumption of work and ensure the stable operation of production lines. Figure 7F shows the information synergy within Cohesion Subgroup 6, which is mainly between government and social organizations, including the Customs Administration, the Civil Aviation Administration, and schools. According to corresponding reports, this subgroup focuses on the problem of returning Chinese people who are stranded in epidemic-stricken countries and regions due to reasons such as studying, working, tourism, or visiting relatives. Figure 7G shows the information synergy within Cohesion Subgroup 7, mainly between government and medical information subjects. From the figure, it can be seen that the Fangcang Hospitals are at the center of information synergy. In Stage T3, Fangcang Hospitals are still an important measure to deal with the epidemic. According to corresponding reports, the information synergy objective of this subgroup is the same as that in Stage T2, which is to ensure the normal operation of Fangcang Hospitals. Figure 7H shows the information synergy within Cohesion Subgroup 8, mainly for information synergy within government information subjects. The National Health Commission and the State Council have played important coordinating roles in overall epidemic prevention and control, and information synergy between the two departments is also very frequent.

Based on the above analysis, it can be seen that in Stage T3, the overlap of node attributes in different cohesion subgroups is still high, but the internal structural complexity has been reduced compared to the previous stage. Information synergy mainly focuses on Cohesion Subgroups a, e, and g, and the focus of information synergy has also shifted, mainly to economic and medical activities, with the main purpose being to restore economic and social order.

### 5.4. Time period T4

**Figure 8:**
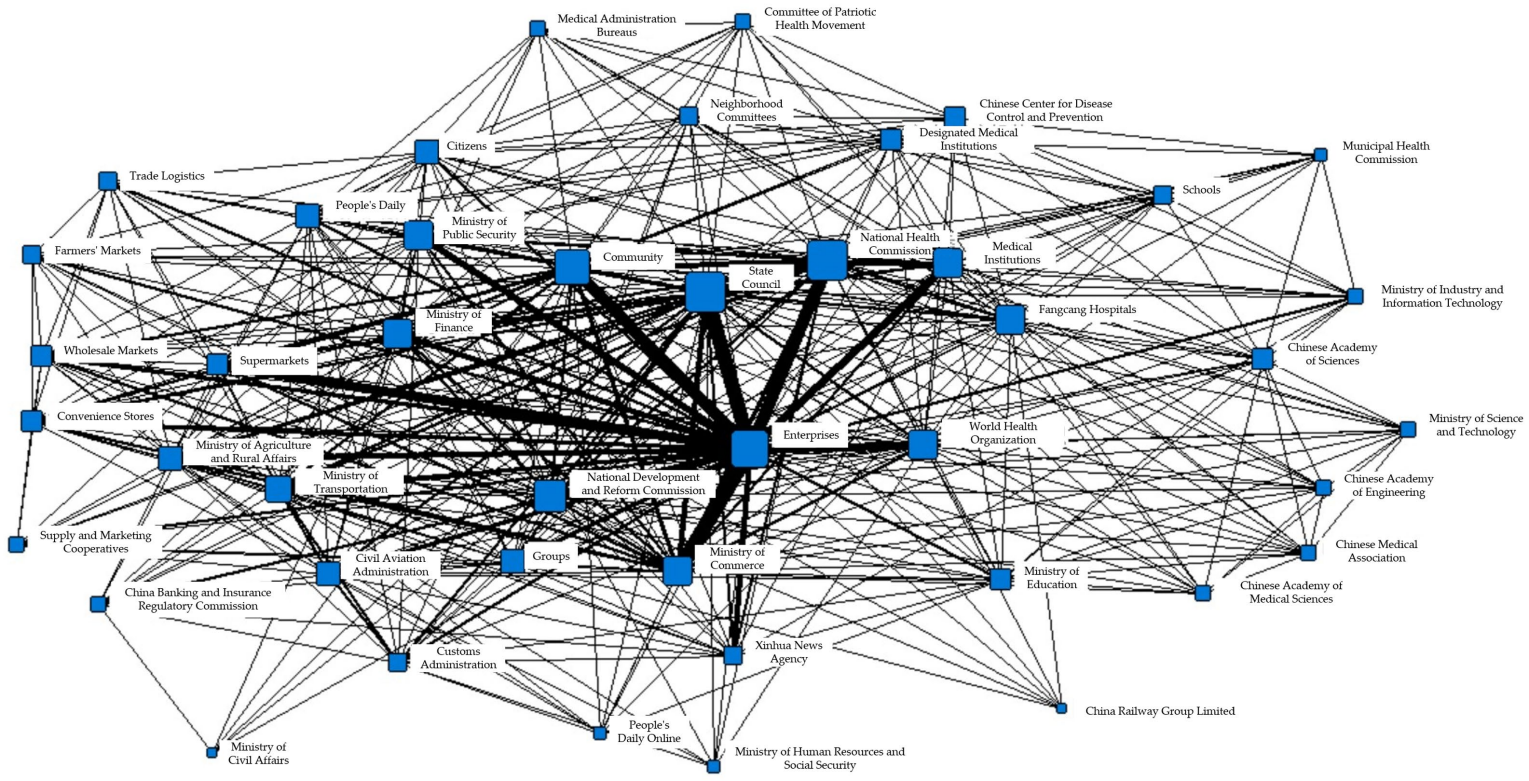
Collaborative network of epidemic information in T4 period.

The total number of T4 collaborations was 27,426, with 44 search terms appearing as shown in the figure above. Among them, the collaboration frequency between enterprises and the State Council was 1,358, and that between enterprises and the National Health Commission was 1,192. The specific information collaboration networks are shown in Figure 8.

Then, the concor analysis algorithm in the Ucinet software was used to analyze the overall information collaboration structure and obtain the following 8 subgroups, as shown in Table 6.

**Table 6:** Classification of cohesive subgroups in T4 period.

Cohesive Subgroup	Search Terms
1	Committee of Patriotic Health Movement, Chinese Center for Disease Control and Prevention, Medical Institutions, Designated Medical Institutions, Health Management Bureaus, Community Committees.
2	Municipal Health Commission, Ministry of Industry and Information Technology, schools.
3	State Council, National Health Commission.
4	Ministry of Education, World Health Organization, Chinese Academy of Sciences, Ministry of Science and Technology, Chinese Academy of Engineering, Chinese Medical Association, Chinese Academy of Medical Sciences.
5	Supermarkets, National Development and Reform Commission, Supply and Marketing Cooperatives, Ministry of Agriculture and Rural Affairs, convenience stores, Ministry of Commerce, Agricultural Markets, Ministry of Transport, Wholesale Markets.
6	Communities, Ministry of Finance, Ministry of Public Security, Citizens.
7	Ministry of Human Resources and Social Security, People’s Daily Online, Ministry of Industry and Information Technology, People’s Daily, Ministry of Civil Affairs, China Banking and Insurance Regulatory Commission.
8	Enterprises, Civil Aviation Administration of China, Customs Administration.

**Figure 9:**
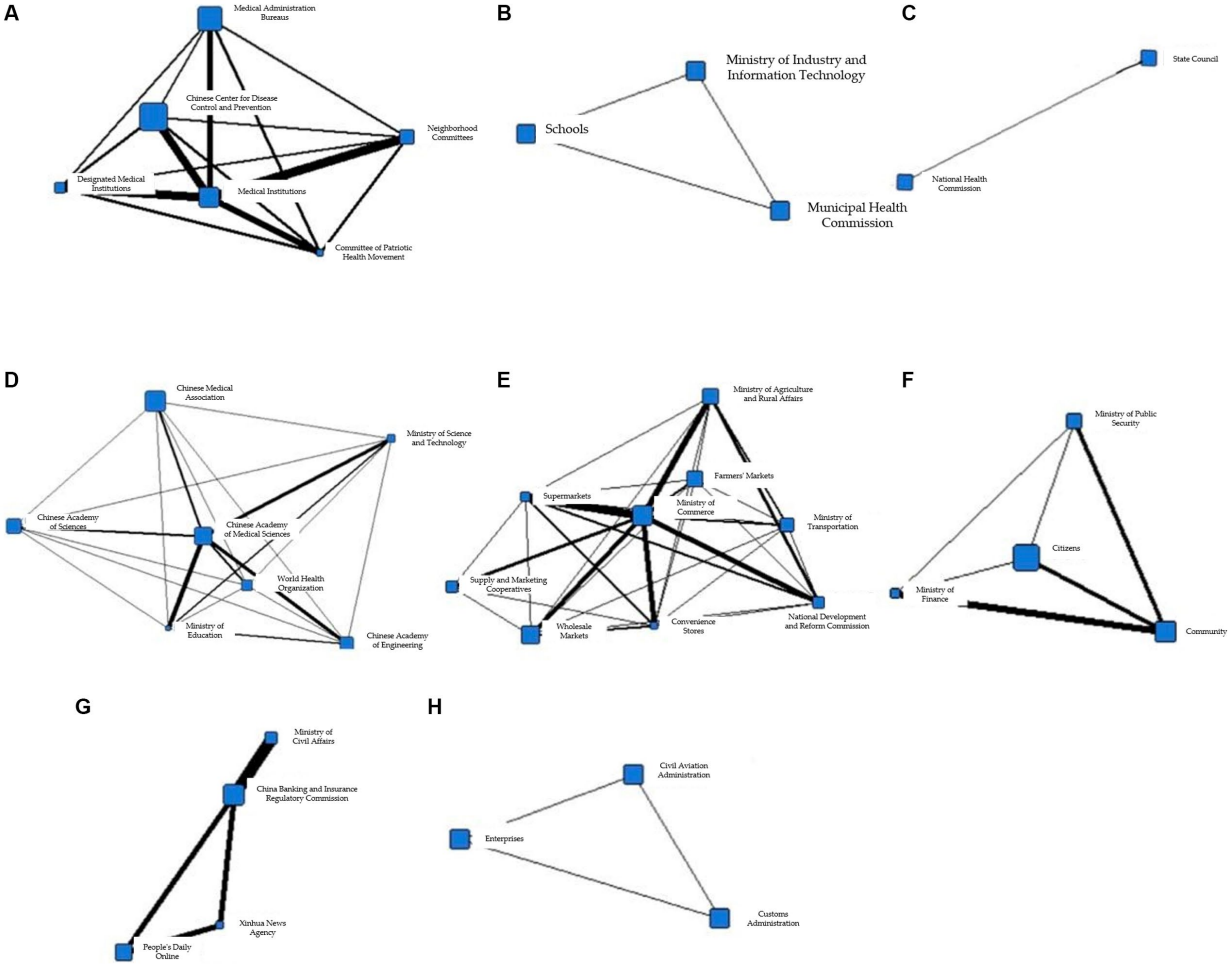
**(A)** Coherent subgroup 1 information collaboration network diagram during T4 period. **(B)** Coherent subgroup 2 information collaboration network diagram during T4 period. **(C)** Coherent subgroup 3 information collaboration network diagram during T4 period. **(D)** Coherent subgroup 4 information collaboration network diagram during T4 period. **(E)** Coherent subgroup 5 information collaboration network diagram during T4 period. **(F)** Coherent subgroup 6 information collaboration network diagram during T4 period. **(G)** Coherent subgroup 7 information collaboration network diagram during T4 period. **(H)** Coherent subgroup 8 information collaboration network diagram during T4 period.

To better illustrate the internal information collaboration within each subgroup, Gephi was used to transform them into the following network diagrams. Figures 9A–H respectively show the interactions between various departments within the eight subgroups.

Figure 9A depicts the information collaboration within cohesive subgroup 1, which includes government, medical, and public information entities. As shown in the figure, medical institutions are positioned at the center of the information collaboration. According to related reports, the main goal of this subgroup’s information collaboration was to diagnose and treat cases. Figure 9B illustrates the information collaboration within cohesive subgroup 2, which mainly consists of social organizations and government information entities. During this period, schools reopened one after another, and various industries gradually resumed work. The main goal of this subgroup’s collaboration was to ensure the health of people returning to work or school. Figure 9C demonstrates the information collaboration within cohesive subgroup 3, which mainly involves information collaboration within the government information entities. The National Health Commission and the State Council both played important coordinating roles in overall epidemic prevention and control, and there was frequent information collaboration between the two departments. Figure 9D shows the information collaboration within cohesive subgroup 4, which mainly includes government and social organization information entities. Social organization information entities, such as various research institutes, were the main players in this subgroup’s information collaboration, indicating that the main purpose of this subgroup’s collaboration was to research and develop vaccines, medicines, and testing reagents. Figure 9E displays the information collaboration within cohesive subgroup 5, which involves the collaboration between government and market information entities. According to corresponding reports, the main goal of this subgroup’s information collaboration was to restore the circulation of goods and commercial order and to ensure the orderly resumption of work and production in society from the aspects of meteorology and food supply. Figure 9F depicts the information collaboration within cohesive subgroup 6, which includes public and government information entities. The main purpose of their information collaboration was to maintain social order and provide consolation and subsidies to people who suffered losses during the epidemic. Figure 9G illustrates the information collaboration within cohesive subgroup 7, which mainly involves information collaboration within government information entities. Its main collaborative purpose was to support the orderly resumption of work and production from the perspective of financial policies. Figure 9H displays the information collaboration within cohesive subgroup 8, which involves the collaboration between market and government information entities. The main goal of this subgroup’s collaboration was to guard against imported cases and stabilize the international supply chain.

Based on the above analysis, it can be concluded that in phase T4, the types of node attributes within cohesive subgroups continued to decrease compared to the previous phase, and the complexity of internal structure also decreased. Information collaboration shifted from full societal participation to small-scale participation within main entities. At this time, the core of the information collaboration network was within cohesive subgroups a, d, and e, where medical departments and economic activity management departments still occupied a central position. In addition, some research-oriented social organizations also occupied a central position. During this period, the main goal of information collaboration was still to restore circulation and consumption while also taking into account epidemic prevention and control as well as resumption of work and business. Compared to the previous period, the division of labor in economic activities became more detailed, providing support for resumption of work and production from multiple aspects such as meteorology, financial policies, market prices, and supply of daily necessities. Additionally, summarizing the post-epidemic experience and prevention was also one of the focuses of T4 phase information collaboration.

### 5.5. Time period T5

**Figure 10:**
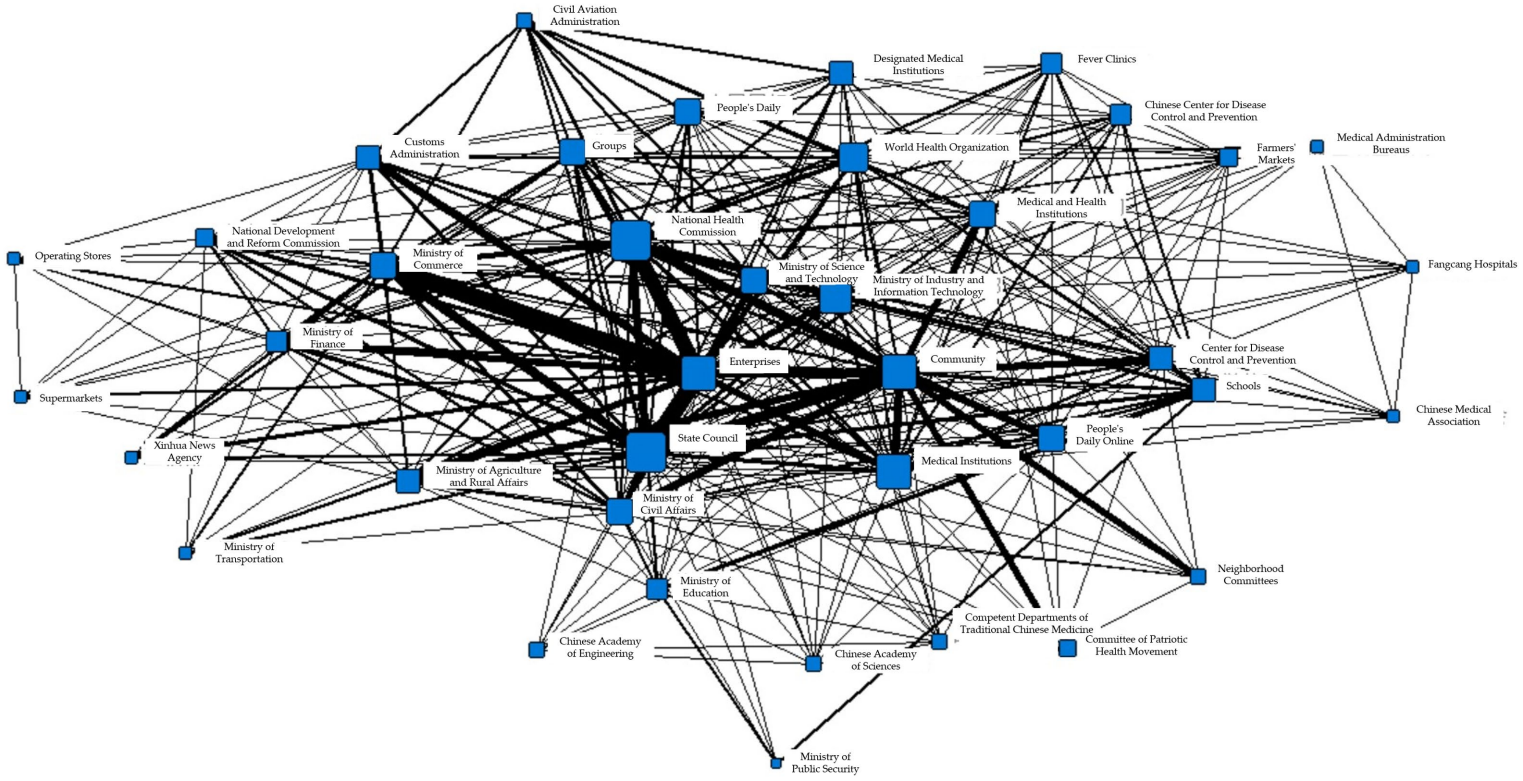
Collaborative network of epidemic information in T5 period.

The total number of collaborations in T5 is 19,738, involving 39 search terms as shown in the above figure. The collaboration frequency between enterprises and the Ministry of Industry and Information Technology is 416, while that between enterprises and the State Council is 340. The specific information collaboration network is shown in Figure 10.

Next, the concor analysis algorithm in the Ucinet software was used to conduct a cohesive subgroup analysis of the overall information collaboration structure, resulting in eight subgroups as shown in Table 7.

**Table 7:** Classification of cohesive subgroups in T5 period.

Cohesive Subgroup	Search Terms
1	Committee of Patriotic Health Movement, Neighborhood Committees, People’s Daily Online, Medical Institutions, Communities.
2	Fever Clinics, Farmers’ Markets, Chinese Center for Disease Control and Prevention, Medical and Health Institutions, World Health Organization, Ministry of Science and Technology, Schools, Designated Medical Institutions, Disease Control Centers, People’s Daily.
3	Health Administration and Medical Management Bureau, Chinese Medical Association.
4	Traditional Chinese Medicine Regulatory Authorities, Ministry of Education, Chinese Academy of Engineering, Chinese Academy of Sciences, Ministry of Public Security.
5	Enterprises, Customs Administration, Ministry of Commerce, Groups, Civil Aviation Administration, Ministry of Finance.
6	Ministry of Civil Affairs, Ministry of Agriculture and Rural Affairs, Ministry of Industry and Information Technology, Ministry of Transport, Supermarkets, Business Stores, National Development and Reform Commission.
7	State Council, National Health Commission.

**Figure 11:**
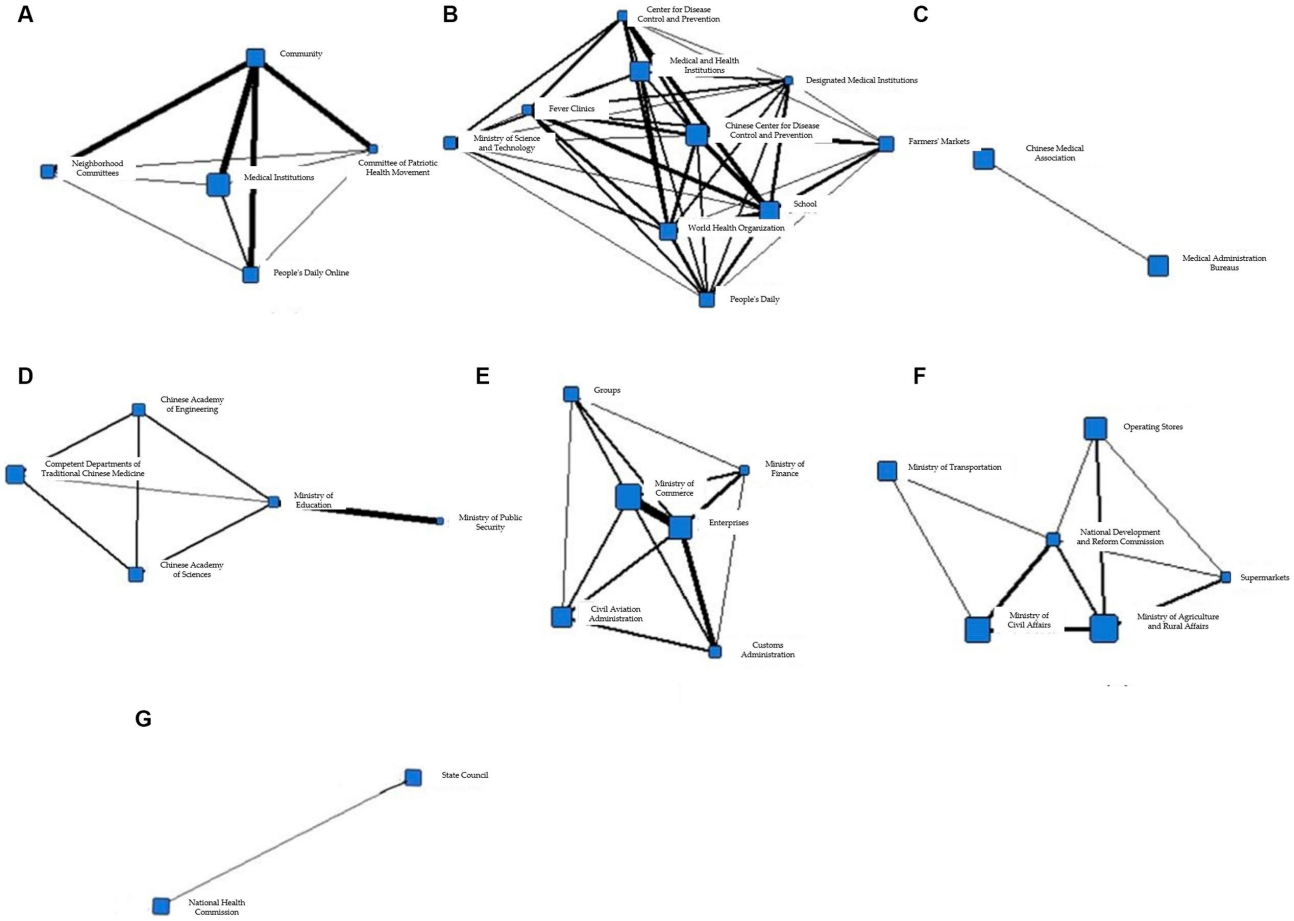
**(A)** Coherent subgroup 1 information collaboration network diagram during T5 period. **(B)** Coherent subgroup 2 information collaboration network diagram during T5 period. **(C)** Coherent subgroup 3 information collaboration network diagram during T5 period. **(D)** Coherent subgroup 4 information collaboration network diagram during T5 period. **(E)** Coherent subgroup 5 information collaboration network diagram during T5 period. **(F)** Coherent subgroup 6 information collaboration network diagram during T5 period. **(G)** Coherent subgroup 7 information collaboration network diagram during T5 period.

To better display the information collaboration within each cohesive subgroup, Gephi was used to transform them into a network structure diagram as shown in Figure 11.

The table in Figure 11A illustrates the information collaboration among the public, medical institutions, and government in Cohesive Subgroup 1. The primary objective of this collaboration is to monitor the health condition of the public and prevent the recurrence of the epidemic. Figure 11B demonstrates the information collaboration among medical institutions, social organizations, markets, and government in Cohesive Subgroup 2. During T5, students resumed classes, and various markets gradually returned to normal operation, which drew considerable attention to schools and markets as gathering places for people. Both Figures 11C,D comprise information collaboration among government and social organizations and their primary objective is the research and application of vaccines and drugs. Figure 11E shows the information collaboration among market and government entities in Cohesive Subgroup 5, which mainly focuses on international economic activities, particularly import and export trade. Figure 11F illustrates the information collaboration between the market and government entities in Cohesive Subgroup 6. The primary objective of information collaboration in this subgroup is to restore the flow of goods and commercial order. Figure 11G displays the information collaboration among government entities in Cohesive Subgroup 7, which includes the internal information collaboration within the government. The National Health Commission and the State Council played a vital coordinating role in overall epidemic prevention and control. Thus, similar to T3 and T4, the information collaboration between these two departments remained frequent during T5.

Based on the above analysis, it can be inferred that the information collaboration during T5 has basically returned to the state similar to that during T1. The information collaboration network is primarily composed of Cohesive Subgroup b, where medical institutions are the core participating entities. By this time, the epidemic has been largely brought under control, and the information collaboration mainly focuses on the medical sector. At the same time, it exists in the form of small-scale entity participation, with clear task division.

## 6. Discussion

**Figure 12:**
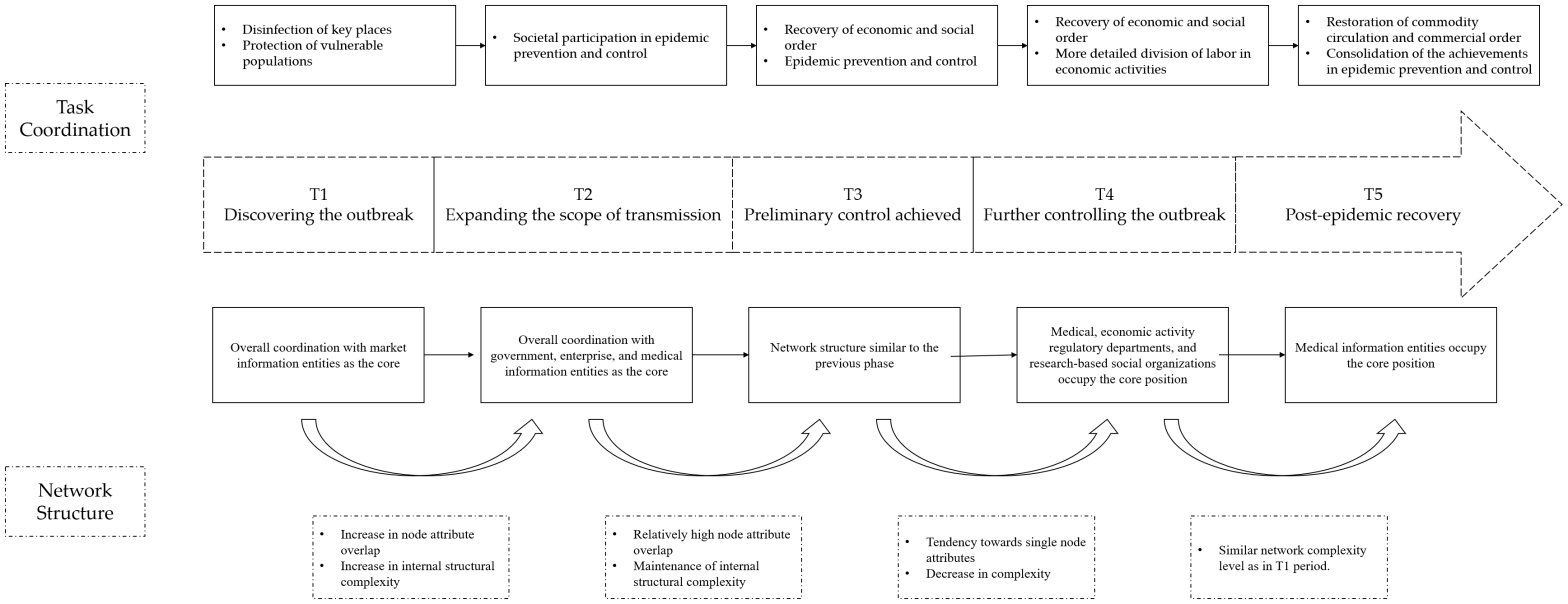
Information collaboration network evolution process diagram.

Through the application of the subgroup coalescing method to analyze the information collaboration structure, collaboration goals, and collaboration paths in different time slices, the overall process of information collaboration evolution for this event has been constructed, as shown in Figure 12.

The initiation of the public health emergency saw information coordination largely limited and typically confined within small networks. Likely due to the early concentration of knowledge and resources within select key institutions, this trend led to localized coordination efforts, a pattern also discernible in preceding public health crises. With the passage of time, the complexity of information networks increased, and the sphere of collaboration extended beyond medical departments, encompassing various societal sectors. This expansion of scope aligns with the consensus in the literature, which states that the increasingly complex nature of health crises necessitates broader sector participation. Some studies assert a consistently high level of societal participation throughout crises; however, the findings of this research indicate a reorientation towards medical departments once the epidemic was under control. This discrepancy could potentially be ascribed to various factors, such as the epidemic’s unique characteristics or the efficiency of the implemented measures, hence warranting further exploration. Furthermore, the pivotal role of government bodies in information collaboration at different stages was observed, reinforcing the importance of robust governmental systems for effective emergency management. The prevalent communication methods between public and governmental information subjects were found to be inefficient despite frequent collaborations, thereby highlighting the need for improvements in existing information collection and transmission systems. This requirement echoes a recurring theme in the literature on public health emergency management. The dynamic nature of collaborative tasks, goals, and methods over time further underscored the necessity for adaptability in information collaboration strategies, a concept widely endorsed.

Examining the evolution of the network structure revealed that the network’s complexity initially increased and later decreased during the public health emergency. As the outbreak progressed, the information collaboration scope broadened to involve various societal sectors. However, once the epidemic was under control, the focus of information collaboration shifted back to medical departments. Throughout this evolution process, diverse information subjects continuously adjusted their information needs to align with the collaboration environment, fostering the evolution of information collaboration. Government information subjects were involved most frequently, and their connections with other information subjects were quite regular, suggesting the urgent need to improve government information collection and transmission systems. The existing mode of communication between public information subjects and government information subjects primarily involves form filling, a time-consuming and inefficient process. Market information subjects confront unique challenges, including dispersed locations and inconsistent information formats, further complicating information collaboration. Therefore, establishing an efficient communication platform among the public, market, and government sectors is pivotal for enhancing information collaboration efficiency during public health emergencies.

Considering the dynamic nature of collaborative tasks during the evolution process, collaborative tasks, goals, and methods evolved over time as the public health emergency unfolded, influencing the network structure and collaboration paths at different stages. Among these tasks, epidemic prevention and control, along with economic recovery, were the most critical throughout the entire information collaboration process. This emphasizes the need to continually adjust the participating information subjects, their collaborative methods, and tasks according to changing collaborative goals. Such adaptability facilitates the construction of a flexible information collaboration mechanism capable of handling cyclical changes in public health emergencies, thus enhancing a country’s emergency management capabilities.

To conclude, this research provides a nuanced understanding of the evolution of information collaboration during sudden public health emergencies. It underscores the importance of future research focusing on enhancing the efficiency of information collaboration, particularly between public and governmental information subjects, and highlights the pivotal role of adaptability in the information collaboration process.

## 7. Conclusion

### 7.1. Potential contributions

Grounded in the theoretical framework of complex adaptive systems, this study presents a novel approach to understanding the dynamics and evolution of information collaboration during public health emergencies. By leveraging rich data generated during the COVID-19 pandemic from January to April 2020, the research provides critical insights into the adaptation and evolution of information collaboration processes in response to the interactions between information subjects and their environments. A salient finding of this study involves the understanding of transformations within information collaboration networks during a crisis. Initial collaboration was concentrated in localized outbreak areas and medical departments, echoing patterns observed in previous health crises. As the crisis escalated, the information collaboration network expanded to encompass a broad array of societal sectors, indicating the involvement of non-medical entities in crisis management. Interestingly, once the epidemic was under control, the focus of information collaboration reverted back to medical departments, suggesting a potential pattern in health emergency crisis management.

Additionally, this research illuminates the critical role of governmental entities in information collaboration at various crisis stages. This finding underscores crisis management literature, highlighting the importance of robust governmental systems for effective public health emergency management. Yet, the study also uncovers certain inefficiencies in the current information communication practices, particularly between public and government information subjects. These findings have the potential to contribute significantly to the refinement and improvement of emergency management policies during public health crises, focusing on enhancing information collaboration’s efficiency and effectiveness.

### 7.2. Limitations and future directions

Despite its contributions, this research acknowledges certain limitations. A primary limitation is the study’s reliance on a single data source, the National Health Commission, which may result in potential omissions of certain aspects of information collaboration, thus presenting an incomplete view of the information collaboration network. Additionally, the analysis is constrained to the early period of the COVID-19 pandemic, which may not encapsulate the full complexity of information collaboration over the course of the entire pandemic.

Future research should, therefore, aim to diversify data sources to provide a more comprehensive and accurate depiction of information collaboration networks during public health emergencies. This may involve utilizing data from various governmental bodies, private sector organizations, and public opinion surveys to capture a broader range of information subjects. Furthermore, research should focus on developing efficient communication platforms connecting public, market, and governmental sectors. Such platforms could potentially address identified inefficiencies in information communication and significantly enhance the efficiency and effectiveness of information collaboration during public health emergencies. The vast potential of digital technology in bolstering information collaboration and crisis management is an area that warrants further exploration in future research.

## Data availability statement

The original contributions presented in the study are included in the article/supplementary material, further inquiries can be directed to the corresponding author.

## Author contributions

MX and KL conceptualized the study. XL and YG curated the data. MX and JS performed formal analysis. KL acquired funding, reviewed and edited the manuscript. MX, XL, and KL developed the methodology. XL and JS validated the results. XL created the visualizations. MX wrote the original draft. All authors contributed to the article and approved the submitted version.
